# Direct Glycan Analysis of Biological Samples and Intact Glycoproteins by Integrating Machine Learning-Driven Surface-Enhanced Raman Scattering and Boronic Acid Arrays

**DOI:** 10.1021/acsmeasuresciau.4c00014

**Published:** 2024-05-15

**Authors:** Qiang Hu, Hung-Jen Wu

**Affiliations:** The Artie McFerrin Department of Chemical Engineering, Texas A&M University, College Station, Texas 77843, United States

**Keywords:** surface-enhanced Raman scattering (SERS), machine learning, boronic acids, glycoprotein, glycan detection, chemometrics

## Abstract

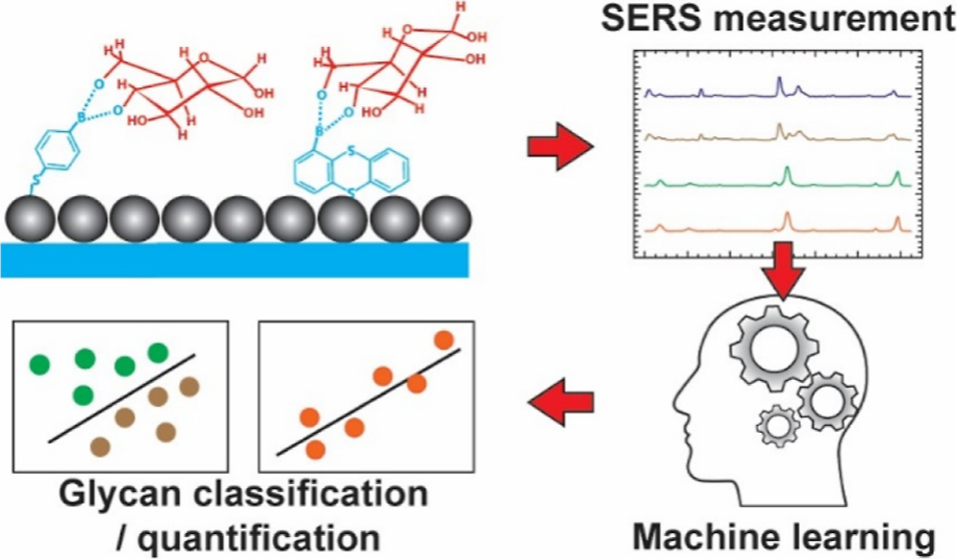

Frequent monitoring of glycan patterns is a critical step in studying glycan-mediated cellular processes. However, the current glycan analysis tools are resource-intensive and less suitable for routine use in standard laboratories. We developed a novel glycan detection platform by integrating surface-enhanced Raman spectroscopy (SERS), boronic acid (BA) receptors, and machine learning tools. This sensor monitors the molecular fingerprint spectra of BA binding to *cis*-diol-containing glycans. Different types of BA receptors could yield different stereoselective reactions toward different glycans and exhibit unique vibrational spectra. By integration of the Raman spectra collected from different BA receptors, the structural information can be enriched, eventually improving the accuracy of glycan classification and quantification. Here, we established a SERS-based sensor incorporating multiple different BA receptors. This sensing platform could directly analyze the biological samples, including whole milk and intact glycoproteins (fetuin and asialofetuin), without tedious glycan release and purification steps. The results demonstrate the platform’s ability to classify milk oligosaccharides with remarkable classification accuracy, despite the presence of other non-glycan constituents in the background. This sensor could also directly quantify sialylation levels of a fetuin/asialofetuin mixture without glycan release procedures. Moreover, by selecting appropriate BA receptors, the sensor exhibits an excellent performance of differentiating between α2,3 and α2,6 linkages of sialic acids. This low-cost, rapid, and highly accessible sensor will provide the scientific community with an invaluable tool for routine glycan screening in standard laboratories.

## Introduction

Glycans are highly abundant biomolecules that can be found in all living organisms. They form dense layers on cell membranes, proteins, and other biomolecules, facilitating a wide range of biochemical reactions.^1^ The glycosylation processes are highly sensitive to various factors, such as environmental conditions, cell activities, nutrition, cell growth cycles, cell health, etc.^2,3^ To investigate glycan-mediated cellular processes, frequent monitoring of glycan changes in biological samples is essential. However, glycan analysis poses significant challenges due to their intricate structures, including complex isomeric forms, glycosidic linkages, and branched structures.

The comprehensive glycan sequencing tools, such as mass spectrometry (MS)-based techniques, are effective but highly resource intensive; therefore, these methods are less suitable for routine use in standard laboratories.^4−6^ Staining samples with lectins (i.e., glycan binding proteins) is another popular tool for comparative glycan analysis. Because the protocol is relatively simple, lectin staining can be applied in standard laboratories.^7^ However, lectin-glycan interactions are not highly specific;^8−11^ a lectin often binds to various glycan structures with different affinities.^12−14^ In addition, the relatively small lectin library could not cover all of the essential glycan structures. Thus, there is a growing demand for a low-cost, rapid, and highly accessible tool that empowers researchers to frequently monitor dynamic changes in glycosylation.^4,6^

**Figure 1:**
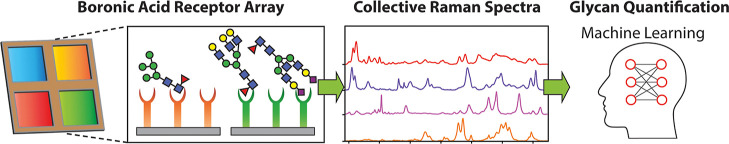
Schematic of the sensor. Glycans are captured by various BAs printed on SERS substrates. Each BA–glycan reaction complex offers a unique molecular fingerprint spectrum. The structural information is enriched by integrating the fingerprint spectra from different BAs, called collective Raman spectra. The complex collective spectra are processed by an advanced ML technique for glycan classification and quantification.

Our prior research developed a machine learning (ML)-driven surface-enhanced Raman spectroscopy (SERS) sensor capable of classifying the selected glycans with remarkable accuracy exceeding 99%.^15^ This new sensing platform includes three major components: (1) boronic acid (BA) receptors, (2) SERS, and (3) ML program (scheme shown in Figure 1). BA can reversibly bind with *cis*-diol-containing carbohydrates, leading to the formation of boronate esters.^16^ It is worth noting that BA binding is not highly specific to a particular glycan structure. Thus, the classic yes/no confirmative response (e.g., fluorescence) in the staining assay is not sufficient to distinguish different glycan structures. To further identify the glycan structures, the sensor monitors molecular fingerprint spectra of the BA–glycan reaction complex.

Raman spectroscopy was chosen to monitor the molecular fingerprint spectra for several reasons. First, Raman spectroscopy can not only provide fingerprint information on molecules but also can distinguish isomeric structures, allowing for isomeric glycan detection.^17^ Second, the availability of low-cost Raman spectrometers will enable widespread adoption.^18^ Additionally, a low-cost plasmonic SERS substrate (<$0.08 per test), called nanopaper, is used to enhance Raman signals.^19,20^

In our previous work, we evaluated two commercially available BA receptors, namely, 4-mercaptophenylboronic acid (4MBA) and 1-thianthrenylboronic acid (1TBA), which effectively captured glycan molecules through *cis*-diol chemical reactions.^15^ Utilizing advanced ML algorithms, we analyzed spectral variations across a wide frequency range for glycan detection. This sensor successfully distinguished stereoisomers and structural isomers featuring different glycosidic linkages.

One of the key observations in our previous study is that the structural information on glycans can be enriched by integrating the spectra obtained from 4MBA and 1TBA. The collective Raman spectra could increase the accuracy of glycan classification and quantification. This discovery offers a strategy to improve sensor performance by using an array of BA receptors. Here, we developed a glycan sensor containing up to 8 different BA receptors to directly analyze the complex biological samples without glycan purification steps, including whole milk and intact glycoproteins. Because SERS is a near-field phenomenon, the resulting Raman signals primarily originate from BA–glycan reaction complexes that are directly adsorbed on metallic nanoparticles. The influences of the background matrices were minimal. As expected, the collective spectra from different BA receptors achieved a remarkable 100% accuracy for classifying the milk oligosaccharides in commercial dairy products.

In addition, we used this platform to directly analyze intact glycoproteins. Protein glycosylation analysis typically requires tedious sample preparation, such as enzymatically releasing glycans from glycoproteins and chemically labeling glycans for detection.^21−23^ Direct analysis of intact glycoproteins will speed up the analysis procedure and benefit the glycobiology community. We evaluated the feasibility of quantifying sialylation levels of the fetuin/asialofetuin mixture. The collective spectra once again demonstrated superior quantification performance, with *R*^2^ and normalized mean square error (NMSE) values of 0.9941 and 0.005912, respectively. Moreover, we evaluated the sensor performance in distinguishing glycosylic linkages of sialic acids. Sialic acid is an important monosaccharide for mammalians due to its functionality in nervous system development, immune regulation, and involvement in many diseases.^24−26^ We tested the sensor’s classification performance on the most common α2,3 and α2,6 sialic acid linkage.^27,28^ By using various BA receptors, the sensor could achieve 100% accuracy in the classification of sialic acid linkages. In summary, this sensor could work as a user-friendly platform to directly detect glycan profiles in biological samples and intact glycoproteins without time-consuming glycan purification steps.

## Methods

### Materials

2-(*N*-Morpholino)ethanesulfonic acid (MES), fetuin, asialofetuin, 2,3-sialyllactose (3-SLA), *N*-acetylneuraminic acid (Neu5Ac/sialic acid), 2,6 sialyllactose (6-SLA), 4MBA, 1TBA, 3-mercaptophenylboronic acid (3MBA), 4-aminophenylboronic acid (4ABA), pyridine-4-boronic acid (PyriBA), pyrene-1-boronic acid (PyreBA), 2-(hydroxymethyl)phenylboronic acid cyclic monoester (HBACM), and benzo[*b*]thien-2-ylboronic acid (BBA) were purchased from Sigma-Aldrich (BA structures are shown in Figure S1). Glass microfiber paper (GF-C, binder free, 100 mm circles) was acquired from Whatman. Silver nitrate (99.99995%), ammonia, dextrose, and 2-propanol were purchased from Thermo Fisher Scientific. All milk products were purchased from local farmers’ markets. All chemicals were of ACS grade or higher and used without further purification.

### Nanopaper Fabrication

Nanopapers were fabricated as previously reported.^15,19,20^ In brief, Tollens’ reagent containing 300 mM ammonia and 50 mM silver nitrate was prepared in a 2 L glass beaker in a 55 °C water bath. Glass microfiber papers were immersed in the solution, and a 500 mm glucose solution was added to initiate the silver mirror reaction. After the reaction was complete, the filter papers were rinsed thoroughly with deionized water and 2-propanol. The resulting products, i.e., the nanopapers, were stored in 2-propanol at room temperature. The storage container was covered with aluminum foil and placed in drawers to prevent light exposure.

### Surface Modification

The surface modification was performed as previously reported.^15^ In brief, nanopapers were cut into a 1 cm × 0.5 cm rectangular shape and then immersed in 50 mM 1TBA or 0.1 mM for other BAs in methanol for 1 h. Before the glycan measurement, the BA-coated nanopaper was air-dried. For the glycan measurement, the BA-coated nanopapers were spotted with the aqueous solutions containing glycans or glycoproteins in 100 mM MES buffer (pH 5) and incubated for 1 h. Before Raman measurement, the paper was dried in an oven at 75 °C for 5 min.

### Raman Measurement

Raman spectra were collected with a Thermo Fisher Scientific DXR3 Raman microscope using laser excitation with a wavelength of 785 nm and an output power of 1 mW. This instrument was equipped with an Olympus BX41 optical microscope and a thermoelectrically cooled charge-coupled detector (Thermo Fisher front-illuminated CCD system) with 1024 × 256 pixel format, operating at −70 °C. The signal was calibrated by an internal polystyrene standard and a 10× objective. The spot size was about 3.8 μm. 200 SERS spectra were collected with an exposure time of 1 s for 5 accumulations at different spots for each sample.

### Milk Oligosaccharide Extraction and Classification

The milk oligosaccharide was extracted using the traditional Folch extraction with slight modification.^29^ Briefly, the milk was mixed with a chloroform and methanol mixture (3:1, v/v) in a 1:4 (milk v/solvent v) ratio in the 50 mL Nalgene Oak Ridge high-speed PTFE FEP centrifuge tubes. The mixture was shaken vigorously for 5 min until homogeneous, followed by 40 min of centrifugation at 4000 rpm. The upper layer of the solution was extracted and concentrated in the rotary evaporator under 55 °C until all solvents were evaporated. Total carbohydrate concentration was determined using the phenol–sulfuric acid assay.^30^ The absorbance of the carbohydrate assay was detected by a microplate reader (BMG Labtech FLUOstar Omega). After that, the oligosaccharide extracts were stored at −20 °C until usage. Three extractions were performed on each milk type on different days.

The dried milk oligosaccharide extracts were reconstituted with DDI water to reach the final total carbohydrate concentration of 0.34 mg/mL, which is the same concentration as the 1 mM lactose solution. The reconstituted oligosaccharide samples were measured under the same protocol as above with 600 spectra collected for each milk type. Before data analysis, the spectra were averaged for three batches, resulting in 200 spectra for each milk type.

### Whole Milk Glycan Profiling

Twenty microliters of cow milk, goat milk, oat milk, soy milk, or almond milk were spotted onto the surface-modified nanopaper using micropipettes and left for 1 h. The paper was then rinsed with 100 mM MES buffer (pH 5) to remove unbound proteins and nonglycan contents. Subsequently, the paper was dried in an oven at 75 °C for 5 min and was measured by a Raman spectrometer using the protocol reported above.

### Intact Protein Quantification

Different ratios of 1 mM fetuin and 1 mM asialofetuin in 100 mM MES buffer (pH 5) were mixed to prepare a titration curve ranging from 0 to 100% of 1 mM fetuin with 20% intervals. The glycoprotein mixtures were spotted on the BA-coated nanopapers and incubated at room temperature for 1 h. The nanopaper was then dried in an oven at 75 °C for 5 min and measured by a Raman spectrometer using the same protocol as above.

### Sialic Acid Linkage Identification

Aqueous solutions of 1 mM 3-SLA, 1 mM 6-SLA, and an equal volume mixture of sialic acid (0.5 mM) and lactose (0.5 mM) were prepared to represent α2,3-sialic linkage, α2,6-sialic acid linkage, and no linkage between sialic acid and other glycans, respectively. The samples were spotted on the BA-coated nanopapers and incubated at room temperature for 1 h. Afterward, the nanopaper was then dried in an oven at 75 °C for 5 min and measured by a Raman spectrometer using the same protocol as reported above, with 200 spectra collected for each concentration.

### Data Processing

The data analysis was performed using the same methodology reported in the previous study.^15^ Briefly, the spectra were first processed using asymmetric least-squares baseline correction. Then, baselined spectra were vector normalized and smoothed using Savitzky–Golay filtering (4th order polynomial, with a frame size of 37). Finally, multivariate analysis techniques and classification algorithms were applied in the spectral range of 400–1650 cm^–1^. Data processing was carried out using MATLAB 2021b.

### Multivariate Analysis and Machine Learning

Prior to applying classifiers, the smoothed spectra underwent multivariate statistical analysis to reduce complexity and extract significant spectral features, explaining the most variance. Discriminant analysis of principal component (DAPC) was used for this purpose.^31^ Principal component analysis was initially applied to reduce the data complexity, and then, a supervised analysis process, discriminant analysis, was used to further discriminate the data set by correlating data variation with the sample information.

After feature extraction, common ML classifiers were used to classify the SERS spectra. A support vector machine was selected due to its superior performance in the prior Raman study.^32^ A fivefold cross-validation was conducted to assess the suitability of the classification algorithm.^33^ In brief, the training and validation sets were established by randomly selecting from the Raman spectral data. The training data set was used to generate a classification model, and the model predicted the validation data set to evaluate the performance. The cross-validation approach was repeated five times, wherein the validation set consisted of 800 and 480 randomly selected SERS spectra in repetition for the whole milk glycan study and sialic acid linkage study, respectively. The model’s performance was evaluated by using classification accuracies, sensitivity, and selectivity. The collective spectra were constructed by combining the truncated BA spectra (400–1650 cm^–1^). Then, the collective spectra went through the same multivariate analysis and classification algorithm as that of the individual spectra.

Regression analysis was conducted using MATLAB 2021b. The Gaussian process regression model was used to predict the percentage fraction of fetuin within the fetuin/asialofetuin mixture. DAPC was first performed on the data set for the wavenumber from 400 to 1650 cm^–1^, and then the resulting canonicals were used in regression analysis. A fivefold cross-validation was performed on the model to evaluate the regression performance. The model was evaluated based on the NMSE and coefficient of determination (*R*^2^). For the collective spectral regression, the data set was built in the same way as described in the classification. Then, the spectra went through the same regression algorithm and were evaluated based on the same performance metrics (NMSE and *R*^2^).

### Statistical Analysis

The data analysis was performed using the same methodology reported in the previous study.^15^ For classification tasks, the performance was evaluated by accuracy, sensitivity, and selectivity. The classification accuracy, sensitivity, and selectivity are defined as
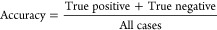
1
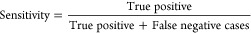
2
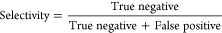
3For quantitative analysis, the performance was evaluated based on the NMSE and *R*^2^
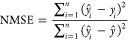
4
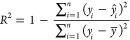
5where  is the predicted values, *y*_*i*_ is the actual values in the data set,  is the mean of the predicted values, *y̅* is the mean of the actual values, and *n* is the number of spectra in the data set.

## Results and Discussion

### Direct Analysis of Unprocessed Milk Samples

Our previous study demonstrated that the ML-driven SERS platform exhibits exceptional performance on identifying purified glycans.^15^ However, the additional purification process is time-consuming and may result in the loss and degradation of the glycans. A new approach allowing the direct analysis of unprocessed biological samples and intact glycoproteins will benefit scientific communities. For the proof-of-concept, we first evaluate the feasibility of analyzing milk oligosaccharides in unprocessed milk samples.

Milk oligosaccharides are pivotal nutrients in human health.^34^ For example, sialic acids in milk are critical for supporting infant body development.^35,36^ Researchers commonly use liquid chromatography-mass spectrometry (LC-MS) to profile *N*-glycans in milk.^34,37^ However, the tedious sample preparation and time-consuming testing process limit their widespread use. In our previous study, we demonstrated that the integration of BAs, SERS, and the ML program could identify and quantify oligosaccharides extracted from milk. Our approach offers a valuable platform for detecting milk adulteration. However, the oligosaccharide extraction process is time and labor-intensive. We are now taking a step further to investigate the feasibility of detecting unprocessed milk samples. Direct detection not only reduces the processing time but also eliminates experimental variations during the oligosaccharide extraction process.

**Figure 2:**
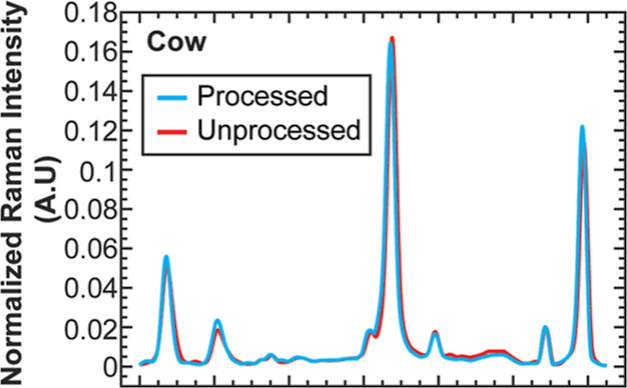
Average normalized SERS spectra (*n* = 200) of the unprocessed cow milk sample and the purified milk oligosaccharides on 4MBA-coated substrates. The milk oligosaccharides were extracted from cow milk using the Folch method. The spectral difference between the processed and unprocessed samples is minimal.

Because SERS is a near-field effect, we hypothesize that the major Raman signals are contributed by BA–glycan reaction complexes that directly attach to SERS substrates. To verify the hypothesis, we compared the SERS spectra of extracted oligosaccharides and unprocessed milk samples on 4MBA-functionalized substrates (the cow milk example is shown in Figure 2 and a detailed comparison among other milk samples is shown in Figure S2). Milk was dropped onto a surface-modified nanopaper, and then the nanopaper was rinsed with the buffer to remove unbound proteins and non-glycan contents. The spectral difference between the processed and unprocessed milk samples is minimal. The small spectral differences were observed at 470 cm^–1^ (CCC out-of-plane bending), 607 cm^–1^ (CCC in-plane bending), 1075 cm^–1^ (CCC in-plane bending, CS stretching), and 1589 cm^–1^ (CC stretching, CH bending). Since the milk oligosaccharides were extracted using the Folch method, lipids and proteins were removed during the separation process.^38^ These minor spectral variations are likely contributed by the additional glycan compounds, such as glycoproteins and glycolipids, in the unprocessed milk samples. The similarity of SERS spectra between extracted oligosaccharides and unprocessed samples suggests that BA is capable of capturing milk oligosaccharides and the major SERS signals were contributed by BA–glycan reaction complexes, despite the presence of other non-glycan constituents in the background. This discovery allowed us to eliminate the time-consuming glycan extraction procedure in the detection protocol.

After demonstrating that the influence of background molecules is minimal, we evaluated the sensor’s capability of classifying commercial dairy products, including cow, goat, soy, oat, and almond milk. In our previous research, we demonstrated that the collective SERS spectra from different BA receptors could enrich the structural information and improve the classification accuracy.^15^ To improve the sensor performance, we established an array of BA receptors. This array consists of 5 different BAs, including two BAs used in our previous study (4MBA and 1TBA) and three additional BAs (3MBA, 4ABA, and PyriBA). The position difference of the mercapto group (3MBA vs 4MBA), the substitution of the mercapto group with amine (4MBA vs 4ABA), and the incorporation of the pyridine group in PyriBA could result in distinct molecular vibrations, leading to unique Raman spectra of BA–glycan reaction complexes. The Raman spectra and confusion matrices of these BAs are shown in Figures S4–S13. Among these BAs, 4MBA exhibited the best performance but still misclassified one sample from all cases. The collective spectra of 5 BAs illustrate the remarkable 100% classification accuracy (Figure S14).

### Direct Analysis of Intact Glycoproteins

The current techniques for glycoprotein analysis require tedious sample preparation, including enzymatically releasing glycans from glycoproteins and chemically labeling glycans for detection.^21−23^ For example, the glycosidase (PNGase F/PNGase A) could be used to cleave the *N*-glycans from glycoproteins, and then the glycans are purified via hydrophilic interaction liquid chromatography.^39,40^ According to the detection techniques, chemical labeling of the glycans may be required for liquid chromatography (LC) or LC-MS.^40,41^ For the *O*-glycan analysis, the procedure can be more complicated due to the lack of enzymatic cleavage methods. While chemical release approaches exist, there is a risk of glycan structure degradation during the release process.^42^ Therefore, the direct analysis of intact glycoproteins without tedious sample preparation is highly desired. For the proof-of-concept, we selected fetuin and asialofetuin as a model system.^43^ Bovine fetuin is known to contain three *N*-glycosylation sites and five O-glycosylation sites,^44^ while asialofetuin shares the same protein structure and glycosylation sites but lacks terminal sialic acids.^45^ Sialic acid plays a crucial role in the central nervous system and the immune system, making it an essential glycan building block.^24^ Here, we used our sensing platform to directly quantify sialylation levels of fetuin–asialofetuin mixtures without sample pretreatment.

**Figure 3:**
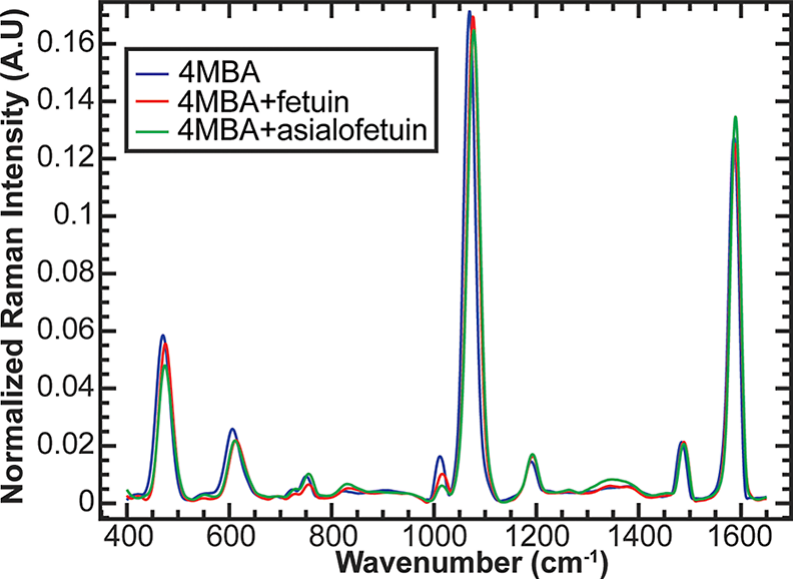
Average normalized SERS spectra (*n* = 200) of fetuin and asialofetuin on 4MBA-coated substrates. Differences could be observed among the 4MBA, 4MBA + fetuin, and 4MBA + asialofetuin spectra.

Figure 3 displays normalized SERS spectra of fetuin and asialofetuin binding to 4MBA, and the spectra of 1TBA, 3MBA, 4ABA, and PyriBA are shown in Figure S15. For 4MBA spectra, sialic acid residues in fetuin cause the peak to shift to a higher wavenumber and the intensity increases at 475 cm^–1^ (CCC out-of-plane bending) and 613 cm^–1^ (CCC in-plane bending). Similarly, the presence of sialic acid residues also results in variations of signal intensities at 1015 cm^–1^ (CC stretching, OH stretching) and at 1589 cm^–1^ (CC stretching, CH bending). These spectral changes are consistent with the data of sialic acid monosaccharides observed in our prior study.^15^ For 1TBA, the spectral differences among fetuin, asialofetuin, and negative control (no glycoprotein) are more significant. With sialic acid residues, the lower spectral signals were observed at 431 cm^–1^ (CCCC torsion, SCCC out-of-plane bending), 669 cm^–1^ (CCCC torsion, CCC in-plane bending), 1037 cm^–1^ (CC stretching), 1125 cm^–1^ (CC stretching, HCC bending), and 1194 cm^–1^ (CC stretching). In contrast, the signals increase at 1081 cm^–1^ (CC stretching) and 1554 cm^–1^ (CC stretching) when sialic acid residues are present. These changes are consistent with our prior observations of sialic acid.^15^

**Figure 4:**
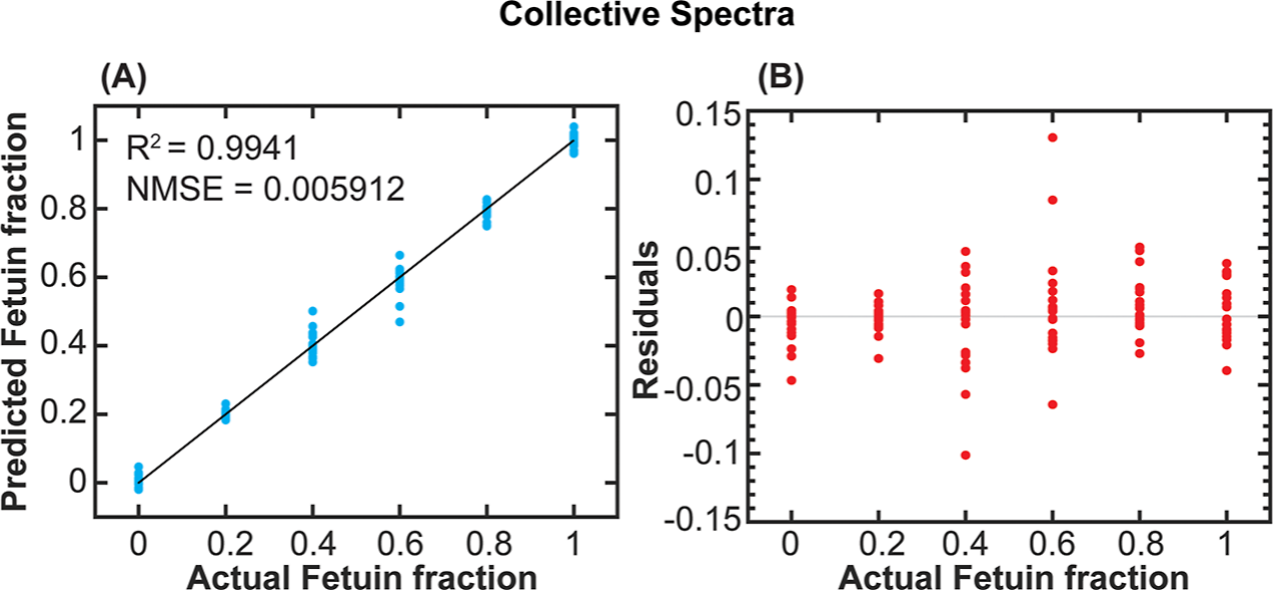
ML regression for the prediction of sialylation levels of fetuin/asialofetuin mixtures. (A) Predicted fetuin fraction vs actual fetuin fraction and the (B) residual plot (residuals vs actual fetuin fraction). The regression was conducted using the collective spectra (4MBA, 1TBA, 3MBA, 4ABA, and PyriBA). The figure shows the quantitative ability of the sensor and demonstrates the capability of directly detecting glycans on intact glycoproteins.

Since the presence of sialic acid residues in SERS spectra was observable, we evaluated the capability of the ML tool to quantify sialic acid levels in the samples containing both fetuin and asialofetuin. Fetuin and asialofetuin were mixed with various molar ratios in a total of 1 mM, and SERS spectra of the mixed samples were collected on the nanopapers functionalized with different BAs. The sialic acid levels were quantified by using Gaussian regression models. Figure S16 illustrates the regression results using 4MBA, while Figures S17–S20 show the results with 1TBA, 3MBA, 4ABA, and PyriBA. Among the five BAs, 3MBA exhibits the best quantification performance, with an *R*^2^ of 0.9920 and NMSE of 0.007982. This is probably because more spectral differences between fetuin and asialofetuin were observed on 3MBA substrates, such as the peak intensity change around 785 and 999 cm^–1^. We also quantified the sialic acid levels using the collective spectra (Figure 4). As expected, the quantification result from the collective spectra of five BA spectra is better than the analysis from the individual BA spectra.

### Sialic Acid Linkage Identification

We have demonstrated that this sensing platform can quantify sialylation levels of intact glycoproteins (mixtures of fetuin and asialofetuin). In our quest for deeper insights, we evaluated the capability of this sensing platform to distinguish sialic acid linkages. Sialic acids could link with other glycan entities in multiple linkage forms (α2,3, α2,6, α2,8, or α2,9),^25,27,28^ and the most common linkages for sialic acids to other glycans are α2,3 and α2,6.^25^ It is crucial to differentiate sialic acid glycosidic linkages since the linkages could influence biological activities. For example, α2,3 linkage may promote selectin binding, and it is related to several cancers and higher patient death rates.^46^ Cancer cells are known to have high levels of α2,6-linked sialic acid with galactose^25^ and α2,6 linkage can block the apoptosis-inducing galectin protein interactions with glycan, which improves cell survival.^27^ The sialic acid linkage also impacts the anti-inflammatory properties of IgG. IgG Fc fragments containing α2,6-linked sialic acid only show a 10-fold increase in anti-inflammatory activity compared to those containing both α2,3- and α2,6-linked sialic acids.^47^ In contrast, Fc fragments containing only α2,3-linked sialic acid show no anti-inflammatory activity at all.^47^ It is crucial to ensure that the IgG antibodies used for anti-inflammatory treatments have the appropriate sialic acid linkages through quality control. IgG could be produced by the HEK or CHO cell lines. However, CHO cell lines only produce α2,3-linked sialic acids while HEK cell lines could produce both types of linkage.^48^ As such, identification of the sialic acid linkage is crucial for quality control in the pharmaceutical industry. However, conventional LC-MS struggles to distinguish between α2,3 and α2,6 linkages since they share the same molecular weight and result in identical *m*/*z* values.^49,50^ Having the ability to distinguish linkages with our platform will significantly benefit the pharmaceutical industry and the glycobiology community.

The stereoselective reactions between BAs and glycans are determined by the spatial orientations and intermolecular distance between BA moieties.^51,52^ The recognition of α2,3 and α2,6 linkages could be improved by selecting the appropriate BA receptors. To explore this, we introduced three new BAs: PyreBA, HBACM, and BBA, alongside the five BAs that are employed in the previous sections (4MBA, 1TBA, 3MBA, 4ABA, and PyriBA) (structures are in Figure S1). PyreBA contains four aromatic rings, known for their distinctive Raman vibrations.^53^ Using PyreBA as a model helps us to understand the role of aromatic ring vibrations in our platform. HBACM has been previously used in detecting monosaccharides, such as glucose and fructose, as well as oligosaccharides like stachyose and nystose.^54,55^ Similarly, BBA closely resembles the structure of HBACM, with the exception of a sulfur atom replacing the oxygen atom.

We examined the performance of the selected BAs in differentiating among the following samples: 3-SLA, 6-SLA, and sialic acid monosaccharide mixed with lactose (SA + Lac) (structural information is shown in Figure S1). 3-SLA consists of a sialic acid residue attached to galactose in a lactose molecule with α2,3 linkage, and 6-SLA has an α2,6-linked sialic acid with lactose. To observe the influence of sialic acid without a linkage, we mixed equal molar concentrations of sialic acid monosaccharides with lactose.

**Figure 5:**
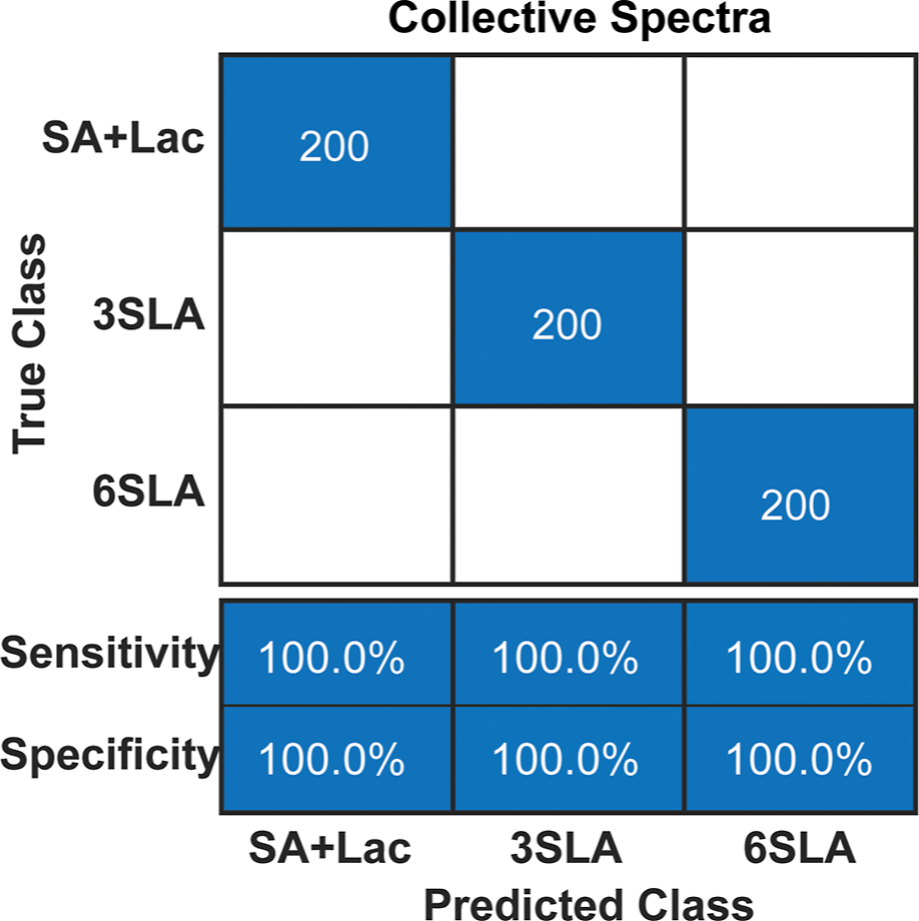
Confusion matrix of sialic acid linkage classification using the collective spectral method (100% accuracy) with all 8 BAs (4MBA, 1TBA, 3MBA, 4ABA, PyriBA, PyreBA, HBACM, and BBA).

Figures S21 and S22 illustrate the confusion matrix and spectra for 4MBA and 1TBA. Both 1TBA and 4MBA successfully distinguished the existence of glycosidic linkages. However, when it came to distinguishing the two different linkages, 4MBA outperformed 1TBA. The difference may be attributed to the poor performance of 1TBA in lactose identification shown in the previous study.^15^ When we analyzed the collective spectra, which incorporated the spectral data from both 4MBA and 1TBA, the accuracy was improved. Figures S23 and S24 showcase the confusion matrix and spectra for 3MBA, 4ABA, and PyriBA, while Figures S25 and S26 present the confusion matrix and spectra for the new BAs, including PyreBA, HBACM, and BBA. Notably, PyreBA did not exhibit strong classification performance. Conversely, both HBACM and BBA delivered satisfactory results compared with the other tested BAs. Of particular interest, 3MBA, 4ABA, and BBA achieved a remarkable 100% accuracy. Finally, Figure 5 shows the confusion matrix for the collective spectra of 8 BAs for sialic acid linkage identification, which resulted in 100% accuracy as well. This discovery demonstrated that the detection performance of the specific glycan structures can be improved by selecting appropriate BA receptors as well as the potential of using up to 8 BAs as an array for glycan profiling.

## Conclusions

We have introduced an ML-driven SERS glycan sensor capable of classifying and quantifying purified glycans with high accuracy.^15^ Expanding upon this foundation, we assessed the platform’s ability to profile glycans in the presence of non-glycan entities. Because SERS is a near-field phenomenon, the Raman signals are majorly contributed by BA–glycan reaction complexes directly attached to the SERS substrates, and the influences of background matrices are minimal. We successfully classified glycans in unprocessed milk samples as well as quantified the sialylation level of intact glycoproteins (a mixture of fetuin and asialofetuin). This discovery allows us to directly analyze biological samples without time-consuming glycan release and extraction procedures. The elimination of sample preparation steps would minimize the loss and degradation of the glycans, eventually reducing experimental variations.

BAs can reversibly bind with *cis*-diol in glycan molecules. By controlling spatial orientations and intermolecular distance between BA moieties, BAs can bind to different pairs of hydroxyl groups on a glycan with different binding affinities.^51,52^ Different types of BAs could yield different stereoselective reactions. In addition, most BAs contain Raman active structures, so the BA–glycan reaction complexes could exhibit unique and strong Raman spectral shifts. We hypothesize that detection accuracy can be improved by integrating Raman spectra collected from different BA receptors. To validate the hypothesis, we established a sensor containing five different BAs to classify milk oligosaccharides and quantify the sialylation levels of fetuin/asialofetuin mixtures. As expected, the detection accuracy was significantly improved by integrating the spectral data obtained from different BA receptors. This result offers a systematic strategy to improve the sensor performance when the complexity of the glycan sample increases.

We also explored how BA structures influence the detection of glycosidic linkages. We examined eight BA receptors containing various functional groups and aryl structures. The appropriate BA receptors, including 3MBA, 4ABA, and BBA, could exhibit excellent performance of differentiating between α2,3 and α2,6 linkages of sialic acids. This discovery suggested that careful selection of BA receptors is crucial to improving the detection accuracy.

In summary, the combination of SERS, BA receptors, and ML-driven chemometrics offers a rapid and efficient approach for comparative glycan detection. This study demonstrated that this sensing platform could directly analyze unprocessed biological samples and intact glycoproteins without glycan purification steps. We also demonstrate that the detection accuracy can be improved by using multiple BA receptors. This sensor can serve as a rapid, low-cost, and valuable tool for routine glycosylation analysis in standard laboratories.

## Supporting Information Available

Chemical structures, SERS spectra, confusion matrix for classification cases, and regression model performance plots (PDF)
