# Supplementary material for: Direct Glycan Analysis of Biological Samples and Intact Glycoproteins by Integrating Machine Learning-Driven Surface-Enhanced Raman Scattering and Boronic Acid Arrays
Source: ACS Meas Sci Au. 2024 May 15;4(3):307–14. doi: 10.1021/acsmeasuresciau.4c00014 (PMC11191725; doi:10.1021/acsmeasuresciau.4c00014)
Supplement: Supplementary file 1 — tg4c00014_si_001.pdf [file tg4c00014_si_001.pdf]

Supplementary Information

Direct Glycan Analysis of Biological Samples and Intact Glycoproteins by Integrating Machine Learning Driven- Surface-Enhanced Raman Scattering (SERS) and Boronic Acid Array

Qiang Hu[1], Hung-Jen Wu[1,*]

[1] The Artie McFerrin Department of Chemical Engineering, Texas A&M University, TX 77843, USA

* Email: hjwu@tamu.edu

Table of Contents

SI Figure 1. Chemical structures for chemicals (boronic acids and glycans) used in the paper. 4

SI Figure 2 Average normalized SERS spectra (n=200) of the unprocessed cow milk sample and the purified milk oligosaccharides (A), unprocessed goat milk sample and purified oligosaccharides (B), and unprocessed soy milk sample and purified oligosaccharides (C) on 4MBA functionalized substrates. The milk oligosaccharides were extracted from milk using Folch method. The spectral difference between processed and unprocessed samples is minimal. 5

SI Figure 3. The difference between average normalized SERS spectra (n=200) of the unprocessed cow milk sample and the purified milk oligosaccharides (A), unprocessed goat milk sample and purified oligosaccharides (B), and unprocessed soy milk sample and purified oligosaccharides (C) on 4MBA functionalized substrates. The milk oligosaccharides were extracted from milk using Folch method. The spectral difference between processed and unprocessed samples is minimal, and the difference between different milk types were obvious. 5

SI Figure 4. Averaged normalized spectra of cow milk, goat milk, soy milk, oat milk, and almond milk on 4MBA functionalized substrates (n=200 for each milk type). 6

SI Figure 5. Confusion matrix of whole milk classification on 4MBA functionalized substrates (99.9% accuracy). 6

SI Figure 6. Average normalized spectra of cow milk, goat milk, soy milk, oat milk, and almond milk on 1TBA functionalized substrates (n=200 for each milk type). 7

SI Figure 7. Confusion matrix of whole milk classification on 1TBA functionalized substrates (99.4% accuracy). 7

SI Figure 8. Average normalized spectra of cow milk, goat milk, soy milk, oat milk, and almond milk on 3MBA functionalized substrates (n=200 for each milk type). 8

SI Figure 9. Confusion matrix of whole milk classification on 3MBA functionalized substrates (99.0% accuracy). 8

SI Figure 10. Average normalized spectra of cow milk, goat milk, soy milk, oat milk, and almond milk on 4ABA functionalized substrates (n=200 for each milk type). 9

SI Figure 11. Confusion matrix of whole milk classification on 4ABA functionalized substrates (98.9% accuracy). 9

SI Figure 12. Average normalized spectra of cow milk, goat milk, soy milk, oat milk, and almond milk on PyriBA functionalized substrates (n=200 for each milk type). 10

SI Figure 13. Confusion matrix of whole milk classification on PyriBA functionalized substrates (99.2% accuracy). 10

SI Figure 14. Confusion matrix on collective spectra (4MBA, 1TBA, 3MBA, 4ABA, PyriBA) of whole milk classification (100% accuracy). 11

SI Figure 15. Average normalized spectra (n= 200) of fetuin and asialofetuin on 1TBA, 3MBA, 4ABA and PyriBA functionalized substrates. 12

SI Figure 16. Predicted fetuin fraction vs. actual fetuin fraction (A) and residual vs. actual fetuin fraction (B) for 4MBA functionalized substrate. 13

SI Figure 17. Predicted fetuin fraction vs. actual fetuin fraction (A) and residual vs. actual fetuin fraction (B) for 1TBA functionalized substrate. 13

SI Figure 18. Predicted fetuin fraction vs. actual fetuin fraction (A) and residual vs. actual fetuin fraction (B) for 3MBA functionalized substrate. 14

SI Figure 19. Predicted fetuin fraction vs. actual fetuin fraction (A) and residual vs. actual fetuin fraction (B) for 4ABA functionalized substrate...14

SI Figure 20. Predicted fetuin fraction vs. actual fetuin fraction (A) and residual vs. actual fetuin fraction (B) for PyriBA functionalized substrates. 14

SI Figure 21. Confusion matrix for sialic acid linkage case (SA+Lac, 3SLA and 6SLA) using 4MBA (99.5% accuracy) and 1TBA functionalized substrates (93.8% accuracy). 15

SI Figure 22. Average normalized spectra (n = 200) for 2,3 sialyllactose (3SLA), 2,6 sialyllactose (6SLA), and sialic acid with lactose (SA+Lac) on 4MBA and 1TBA functionalized substrates. 16

SI Figure 23. Confusion matrix for sialic acid linkage case (SA+Lac, 3SLA and 6SLA) using 3MBA (100% accuracy) 4ABA (100% accuracy) and PyriBA functionalized substrates (100% accuracy)...................17

SI Figure 24. Average normalized spectra (n = 200) for 2,3 sialyllactose (3SLA), 2,6 sialyllactose (6SLA), and sialic acid with lactose (SA+Lac) on 3MBA, 4ABA, and PyriBA functionalized substrates..........18

SI Figure 25. Confusion matrix for sialic acid linkage case (SA+Lac, 3SLA and 6SLA) using PyreBA (96.5% accuracy) HBACM (99.5% accuracy) and BBA functionalized substrates (100% accuracy). 19

SI Figure 26. Average normalized spectra (n = 200) for 2,3 sialyllactose (3SLA), 2,6 sialyllactose (6SLA), and sialic acid with lactose (SA+Lac) on 3MBA, 4ABA, and PyriBA functionalized substrates..........20

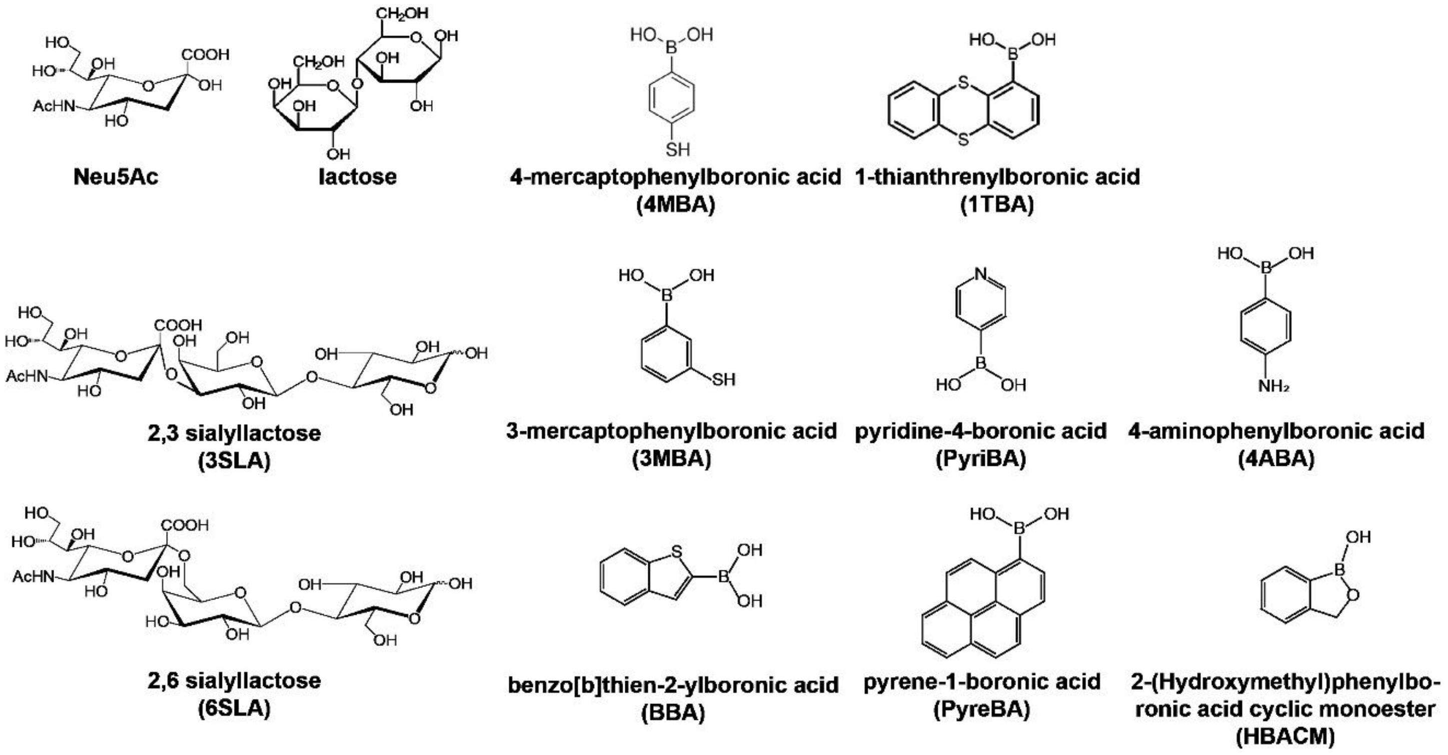

SI Figure 1. Chemical structures for chemicals (boronic acids and glycans) used in the paper.

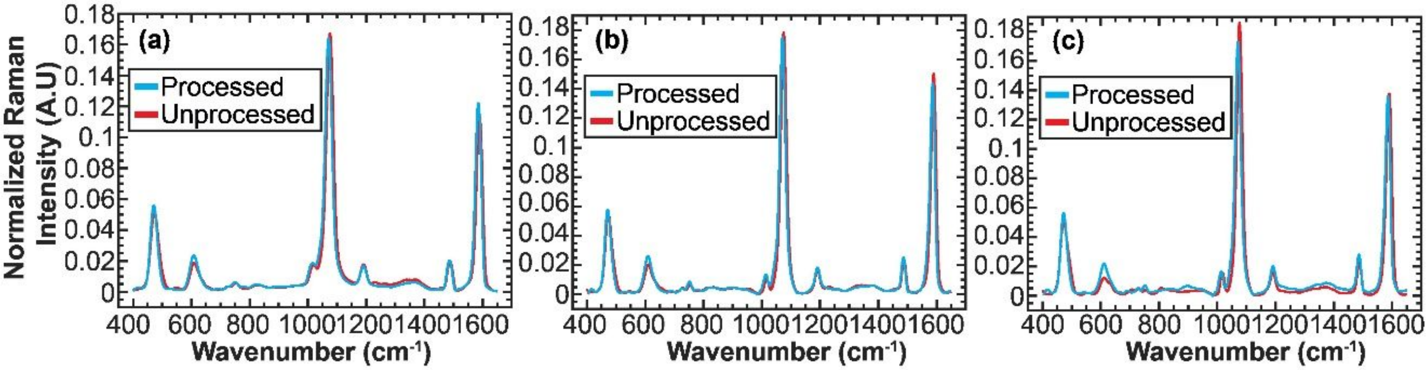

SI Figure 2 Average normalized SERS spectra (n=200) of the unprocessed cow milk sample and the purified milk oligosaccharides (A), unprocessed goat milk sample and purified oligosaccharides (B), and unprocessed soy milk sample and purified oligosaccharides (C) on 4MBA functionalized substrates. The milk oligosaccharides were extracted from milk using Folch method. The spectral difference between processed and unprocessed samples is minimal.

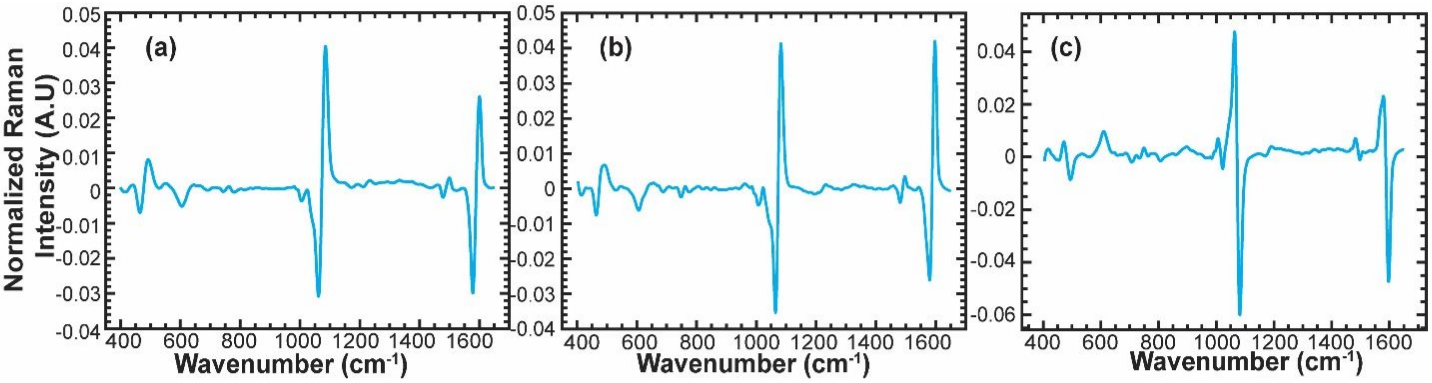

SI Figure 3. The difference between average normalized SERS spectra (n=200) of the unprocessed cow milk sample and the purified milk oligosaccharides (A), unprocessed goat milk sample and purified oligosaccharides (B), and unprocessed soy milk sample and purified oligosaccharides (C) on 4MBA functionalized substrates. The milk oligosaccharides were extracted from milk using Folch method. The spectral difference between processed and unprocessed samples is minimal, and the difference between different milk types were obvious.

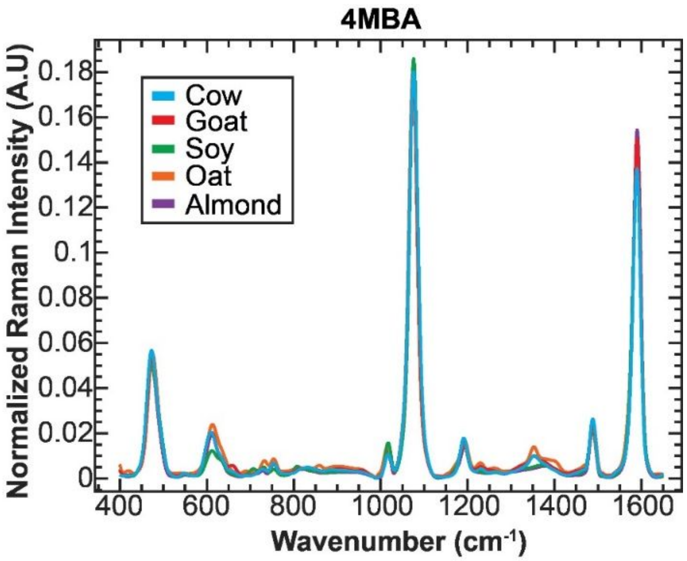

SI Figure 4. Averaged normalized spectra of cow milk, goat milk, soy milk, oat milk, and almond milk on 4MBA functionalized substrates (n=200 for each milk type).

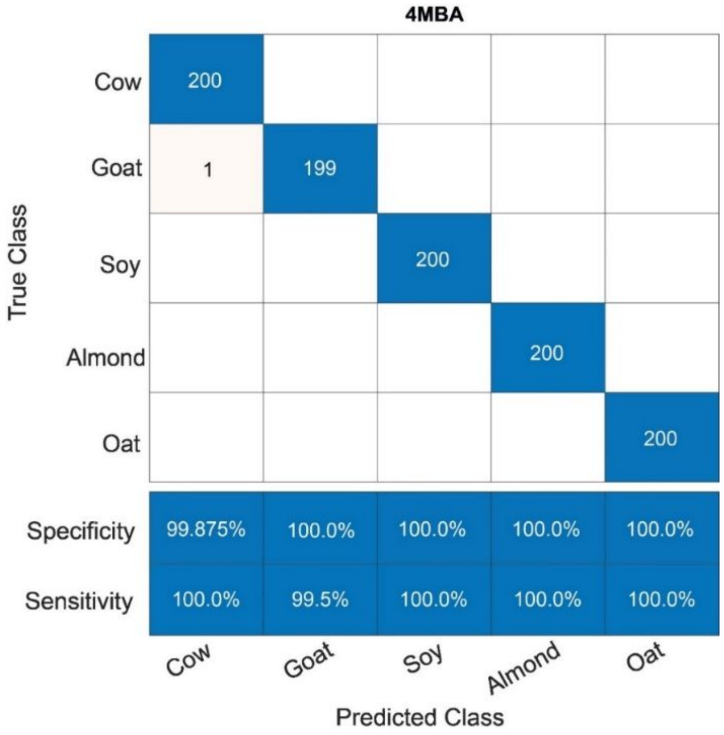

SI Figure 5. Confusion matrix of whole milk classification on 4MBA functionalized substrates (99.9% accuracy).

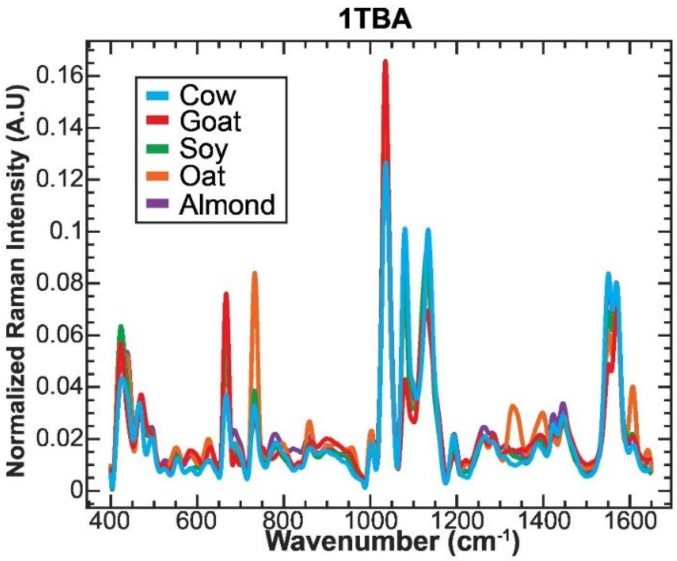

SI Figure 6. Average normalized spectra of cow milk, goat milk, soy milk, oat milk, and almond milk on 1TBA functionalized substrates (n=200 for each milk type).

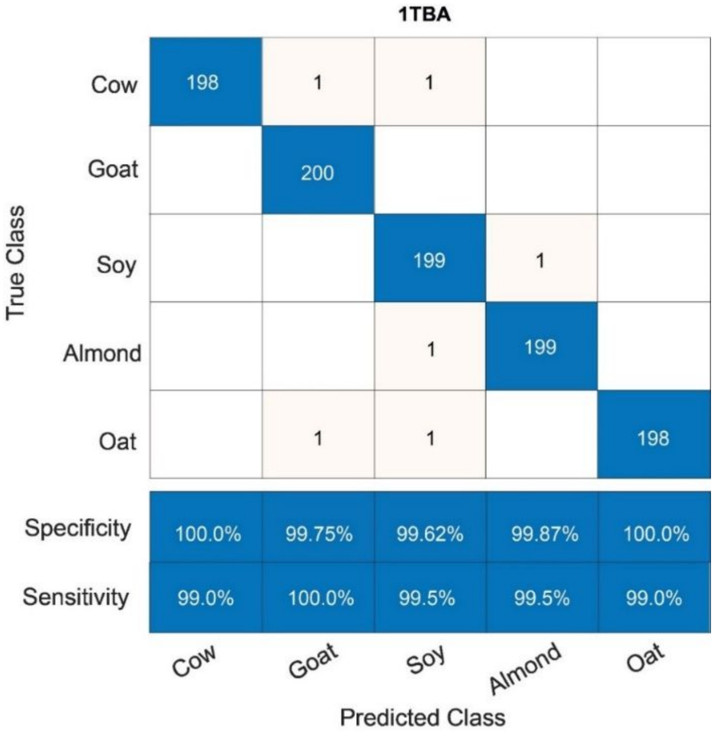

SI Figure 7. Confusion matrix of whole milk classification on 1TBA functionalized substrates (99.4% accuracy).

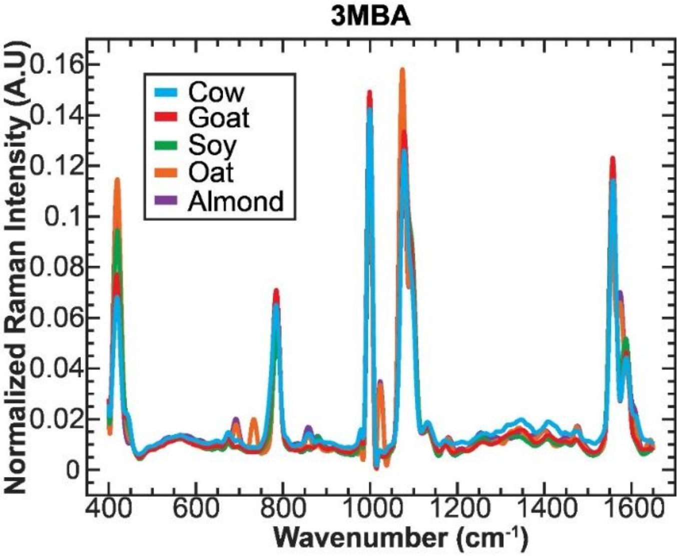

SI Figure 8. Average normalized spectra of cow milk, goat milk, soy milk, oat milk, and almond milk on 3MBA functionalized substrates (n=200 for each milk type).

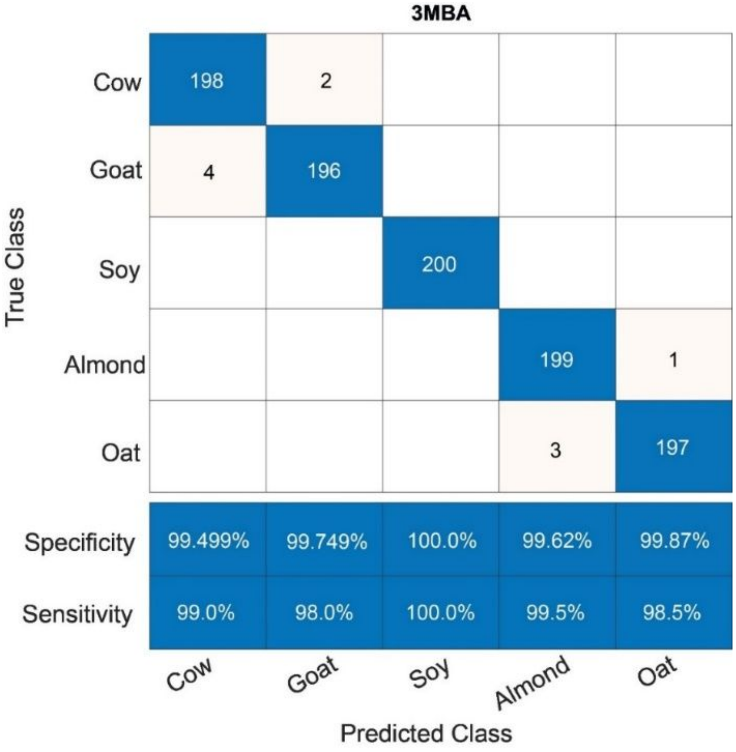

SI Figure 9. Confusion matrix of whole milk classification on 3MBA functionalized substrates (99.0% accuracy).

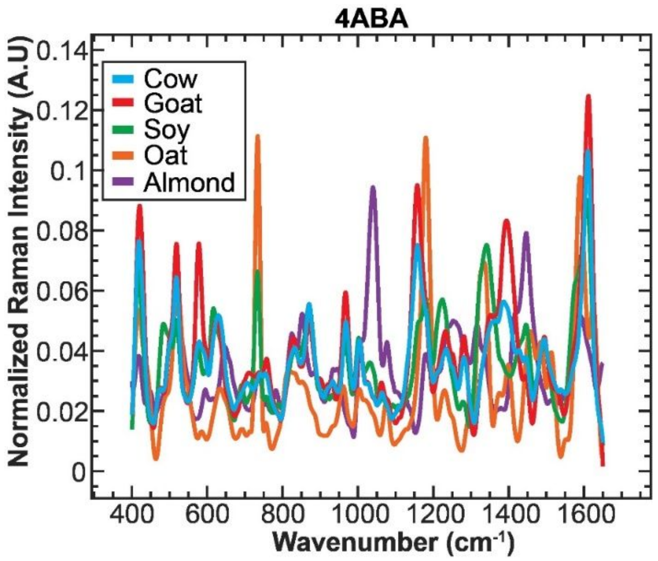

SI Figure 10. Average normalized spectra of cow milk, goat milk, soy milk, oat milk, and almond milk on 4ABA functionalized substrates (n=200 for each milk type).

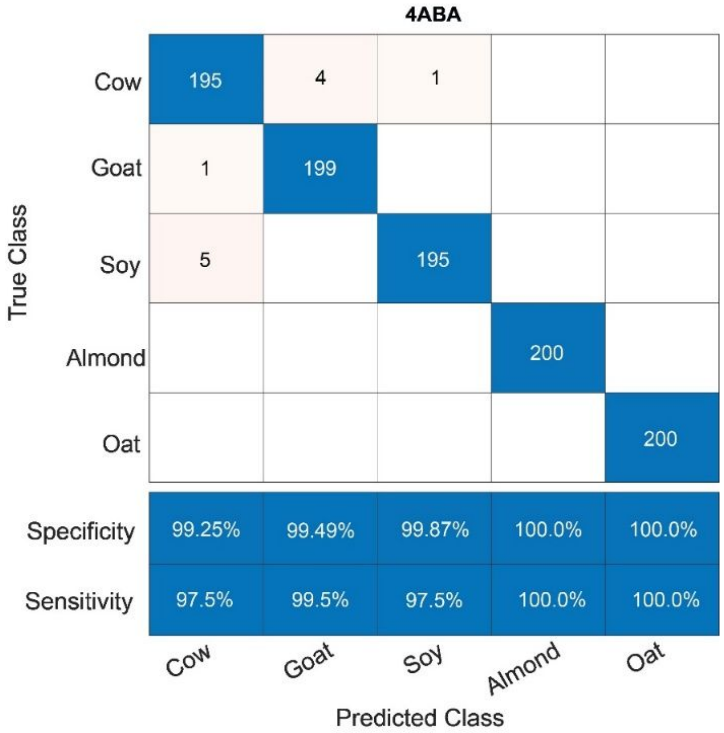

SI Figure 11. Confusion matrix of whole milk classification on 4ABA functionalized substrates (98.9% accuracy).

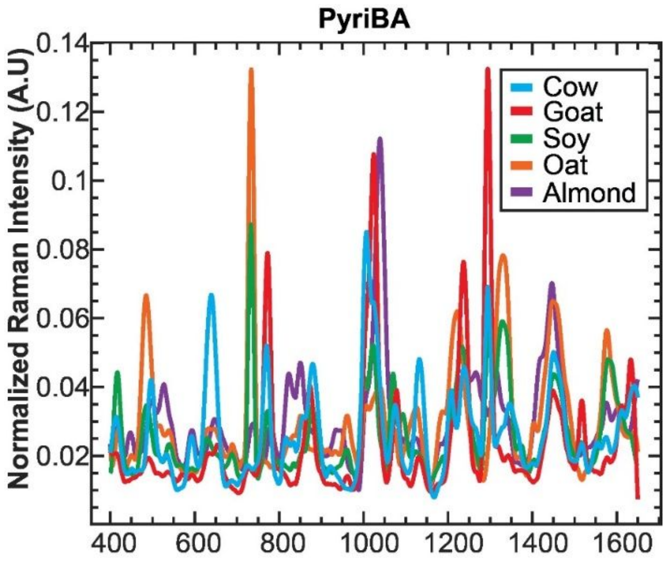

SI Figure 12. Average normalized spectra of cow milk, goat milk, soy milk, oat milk, and almond milk on PyriBA functionalized substrates (n=200 for each milk type).

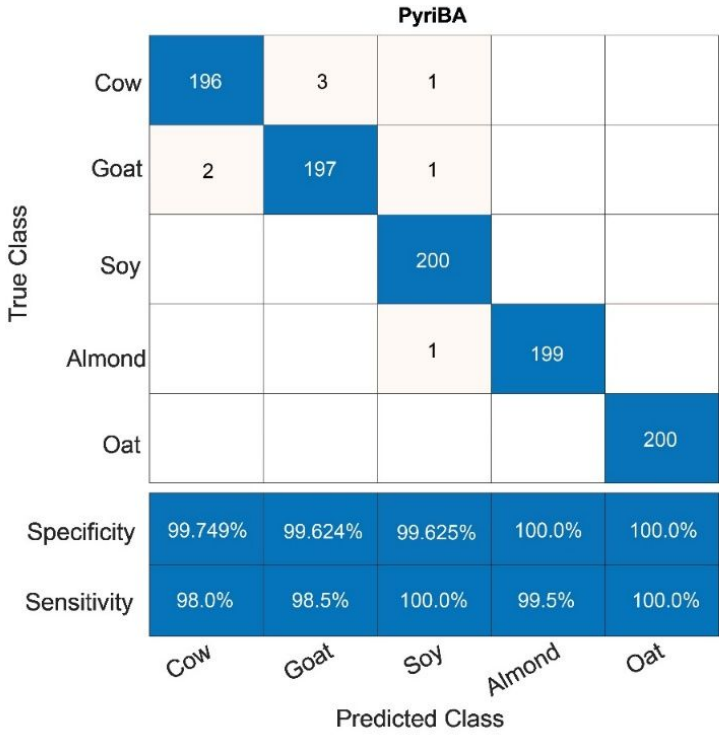

SI Figure 13. Confusion matrix of whole milk classification on PyriBA functionalized substrates (99.2% accuracy).

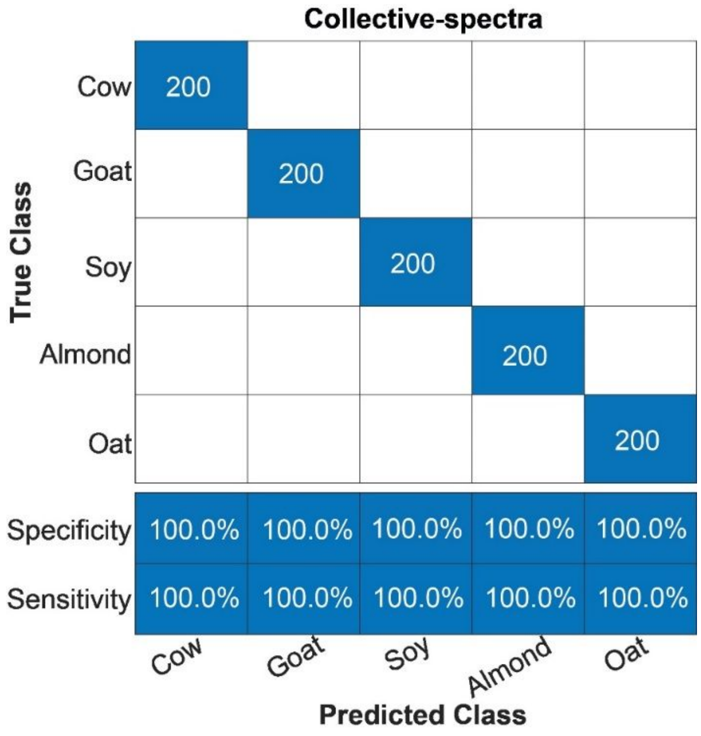

SI Figure 14. Confusion matrix on collective spectra (4MBA, 1TBA, 3MBA, 4ABA, PyriBA) of whole milk classification (100% accuracy).

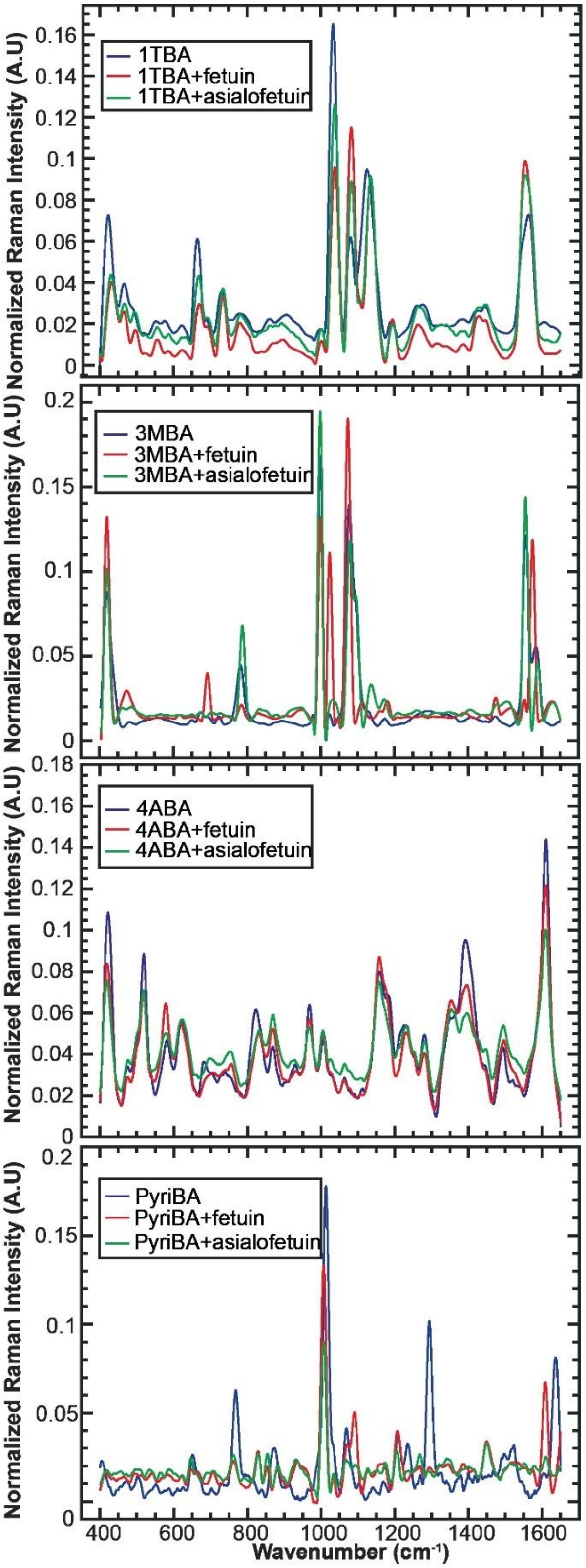

SI Figure 15. Average normalized spectra (n= 200) of fetuin and asialofetuin on 1TBA, 3MBA, 4ABA and PyriBA functionalized substrates.

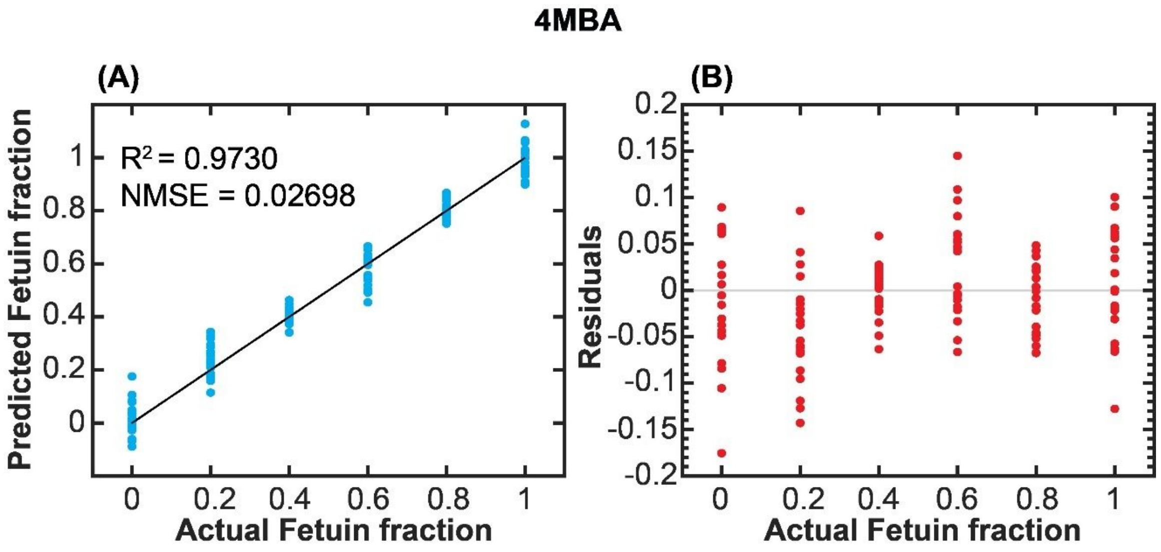

SI Figure 16. Predicted fetuin fraction vs. actual fetuin fraction (A) and residual vs. actual fetuin fraction (B) for 4MBA functionalized substrate.

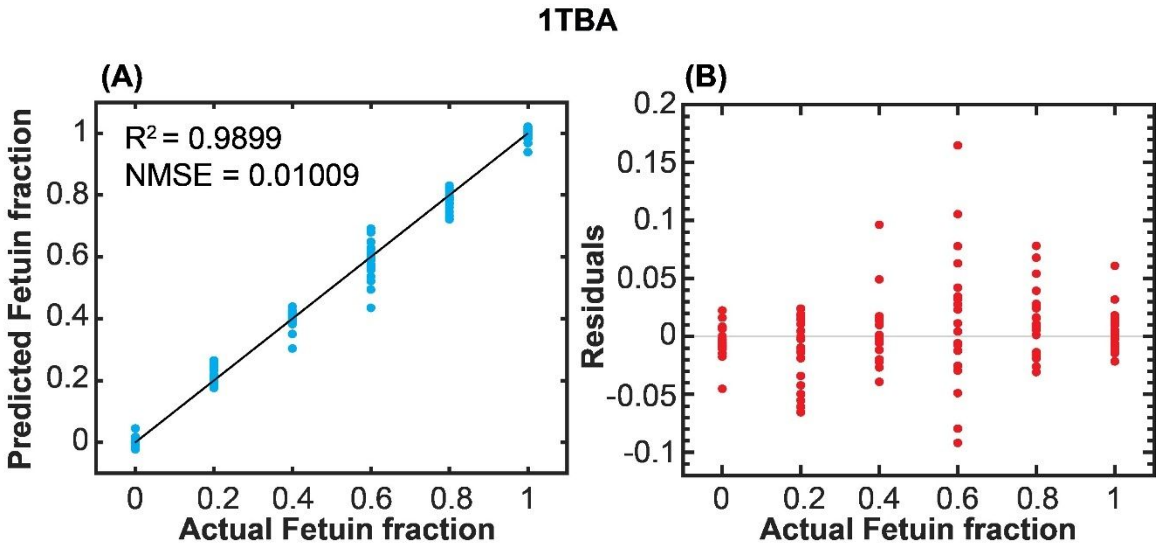

SI Figure 17. Predicted fetuin fraction vs. actual fetuin fraction (A) and residual vs. actual fetuin fraction (B) for 1TBA functionalized substrate.

3MBA

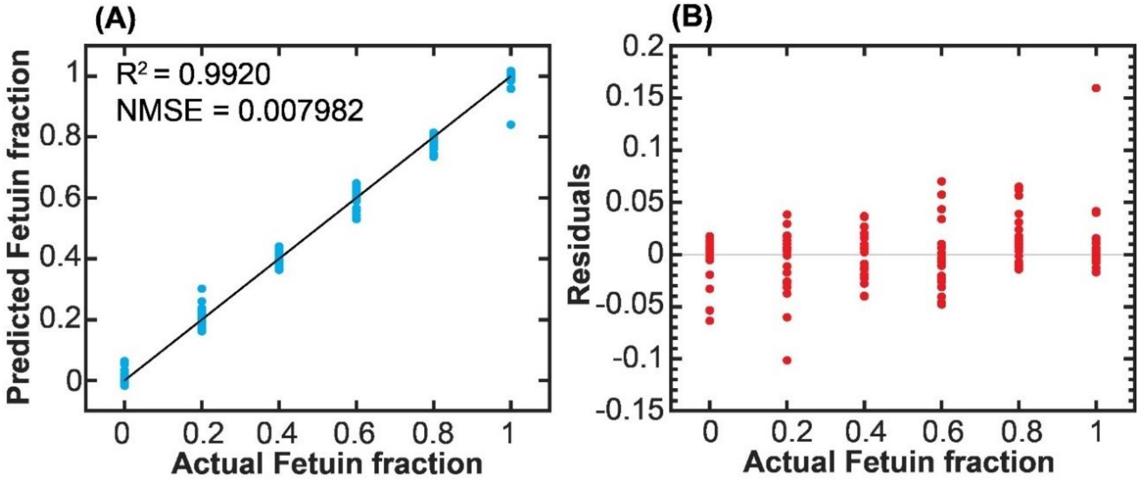

SI Figure 18. Predicted fetuin fraction vs. actual fetuin fraction (A) and residual vs. actual fetuin fraction (B) for 3MBA functionalized substrate.

4ABA

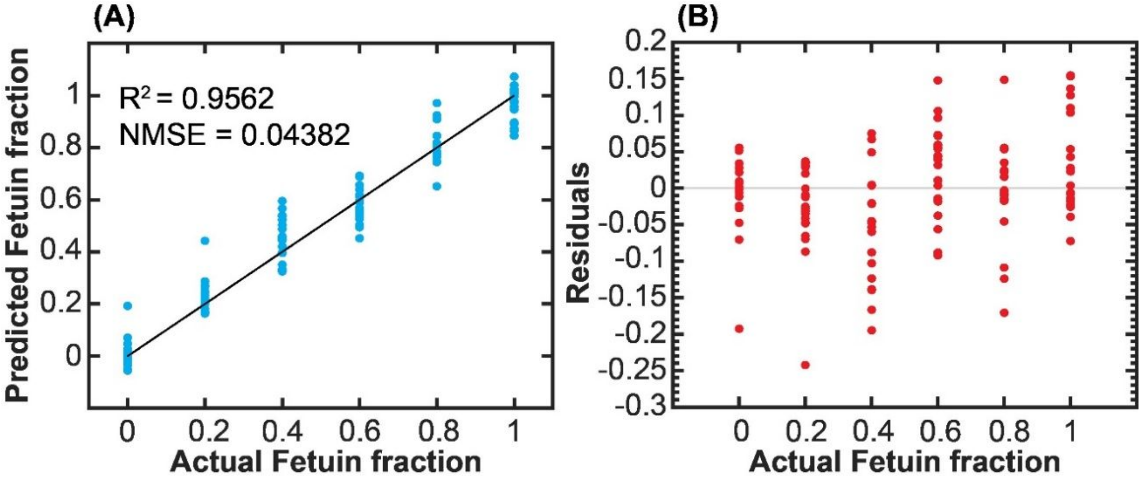

SI Figure 19. Predicted fetuin fraction vs. actual fetuin fraction (A) and residual vs. actual fetuin fraction (B) for 4ABA functionalized substrate.

PyriBA

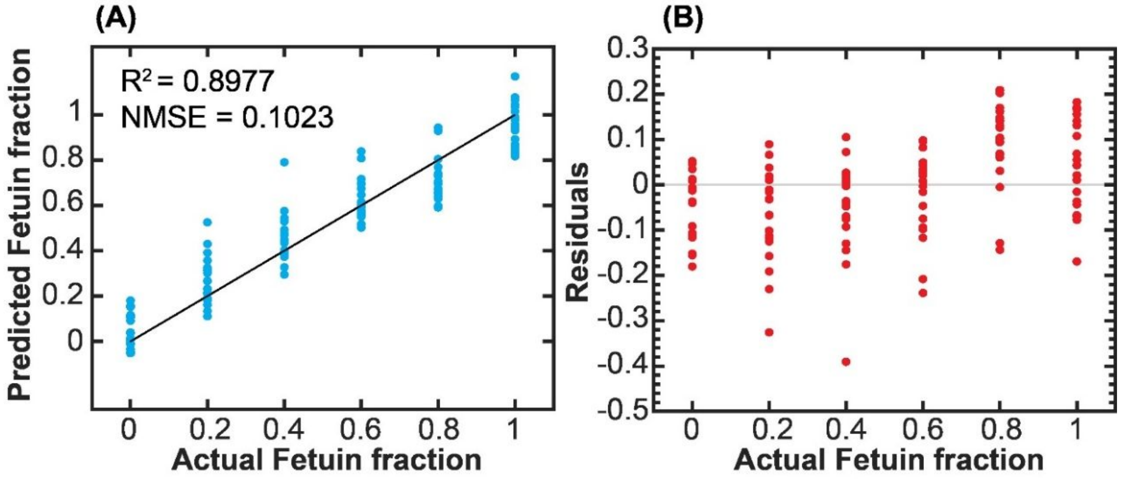

SI Figure 20. Predicted fetuin fraction vs. actual fetuin fraction (A) and residual vs. actual fetuin fraction (B) for PyriBA functionalized substrates.

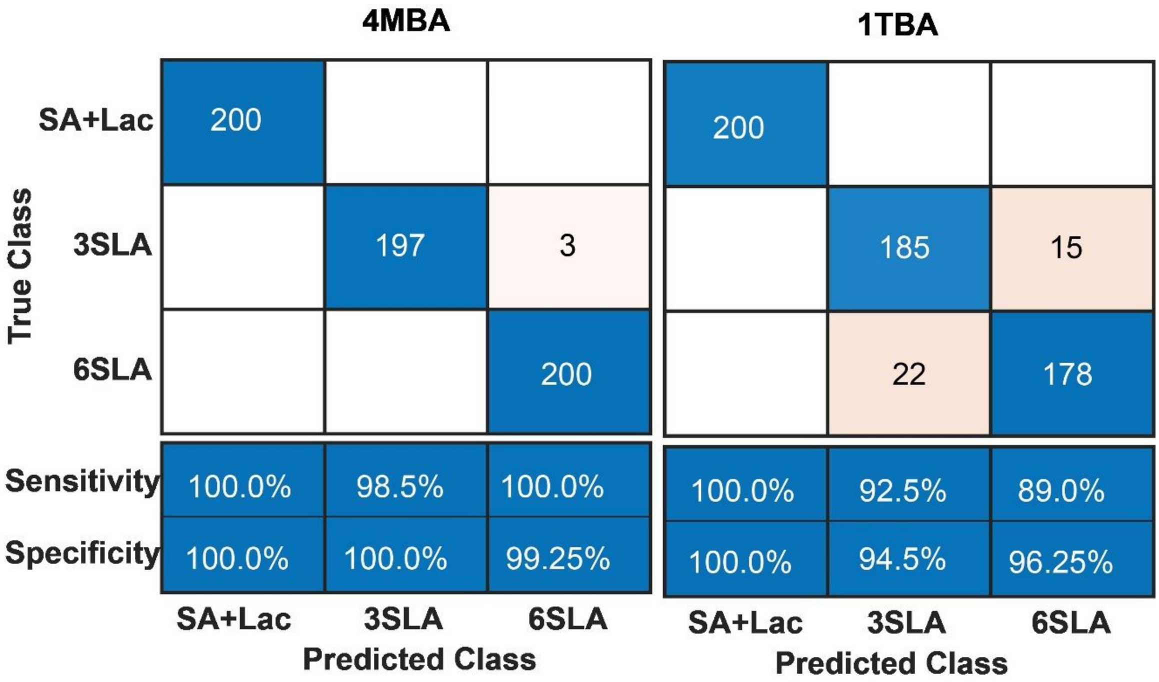

SI Figure 21. Confusion matrix for sialic acid linkage case (SA+Lac, 3SLA and 6SLA) using 4MBA (99.5% accuracy) and 1TBA functionalized substrates (93.8% accuracy).

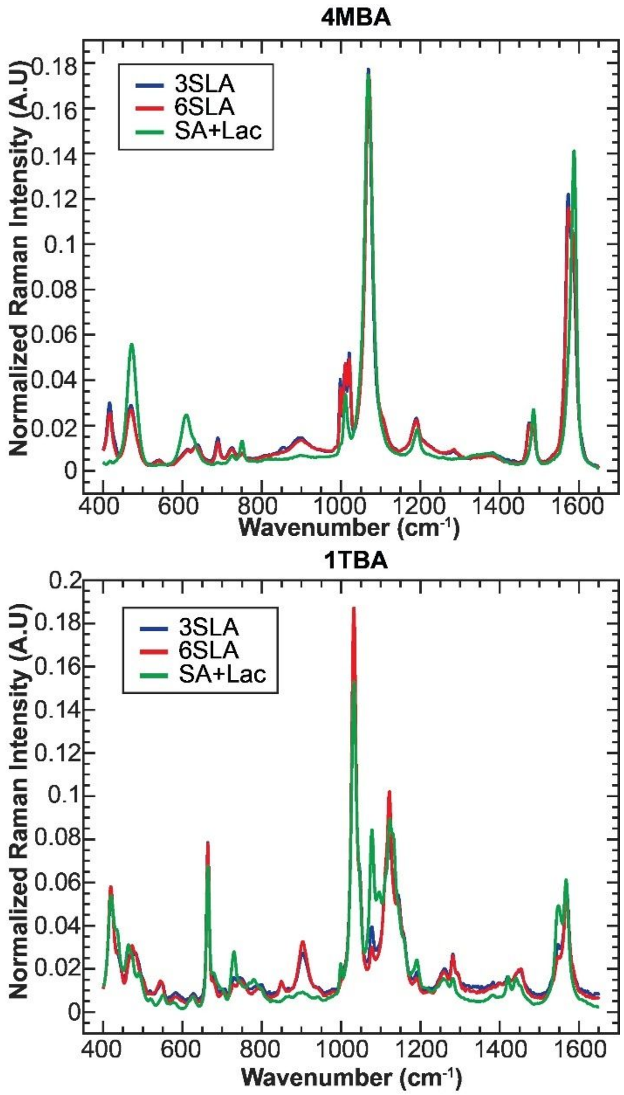

SI Figure 22. Average normalized spectra (n = 200) for 2,3 sialyllactose (3SLA), 2,6 sialyllactose (6SLA), and sialic acid with lactose (SA+Lac) on 4MBA and 1TBA functionalized substrates.

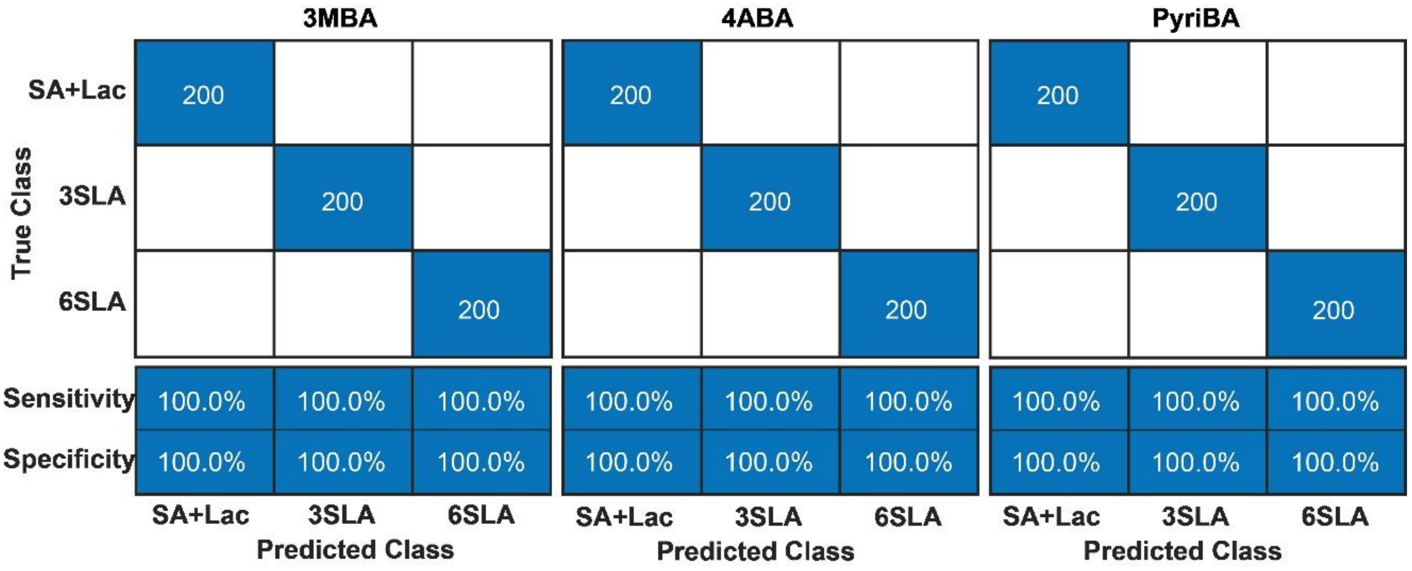

SI Figure 23. Confusion matrix for sialic acid linkage case (SA+Lac, 3SLA and 6SLA) using 3MBA (100% accuracy) 4ABA (100% accuracy) and PyriBA functionalized substrates (100% accuracy).

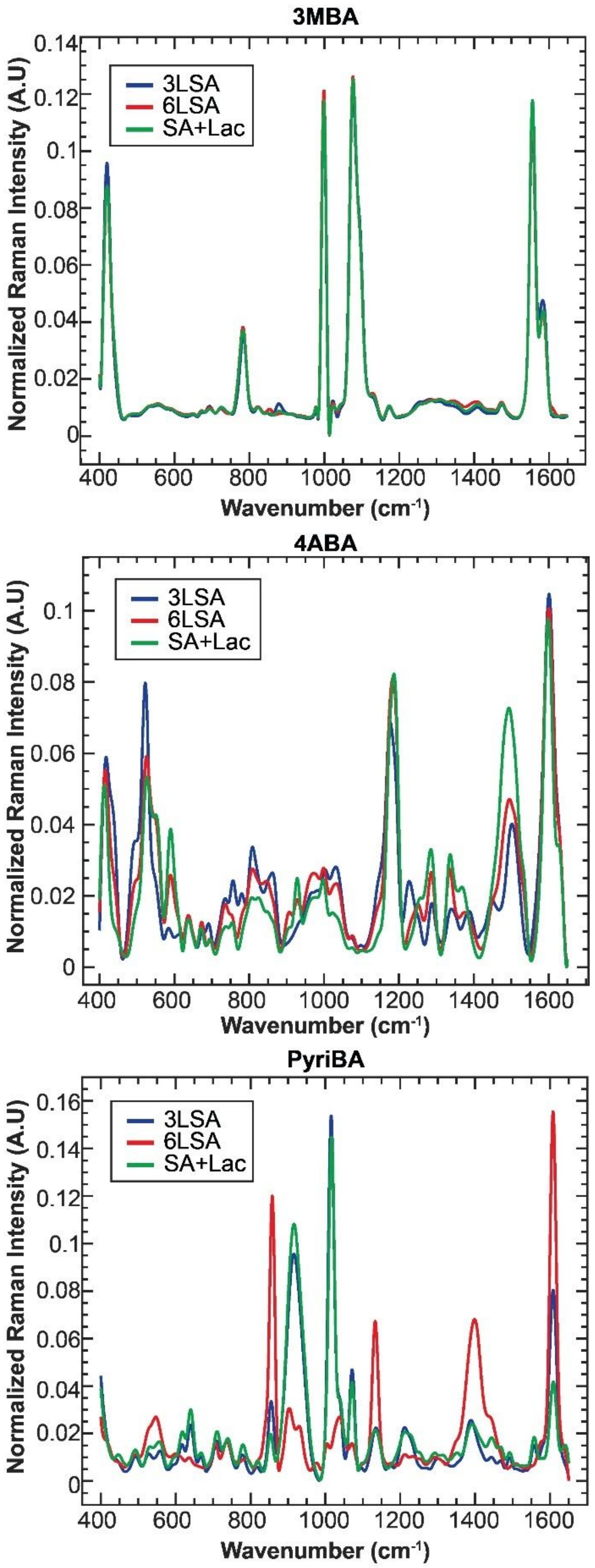

SI Figure 24. Average normalized spectra (n = 200) for 2,3 sialyllactose (3SLA), 2,6 sialyllactose (6SLA), and sialic acid with lactose (SA+Lac) on 3MBA, 4ABA, and PyriBA functionalized substrates.

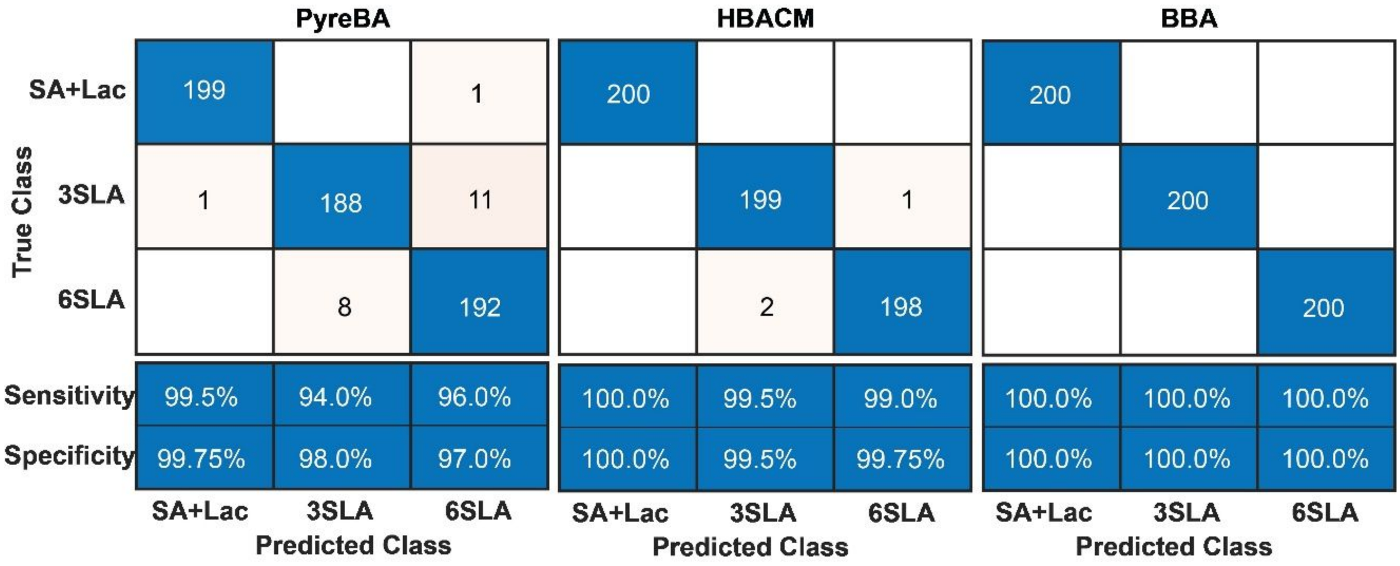

SI Figure 25. Confusion matrix for sialic acid linkage case (SA+Lac, 3SLA and 6SLA) using PyreBA (96.5% accuracy) HBACM (99.5% accuracy) and BBA functionalized substrates (100% accuracy).

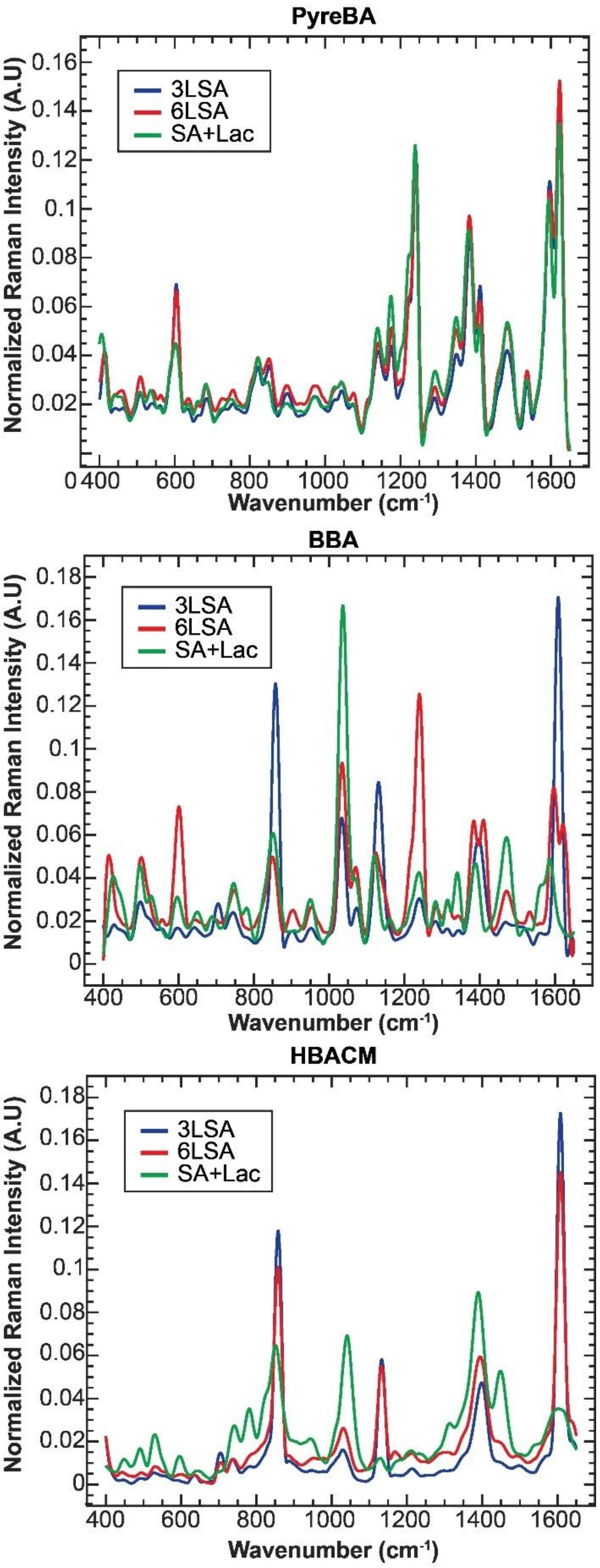

SI Figure 26. Average normalized spectra (n = 200) for 2,3 sialyllactose (3SLA), 2,6 sialyllactose (6SLA), and sialic acid with lactose (SA+Lac) on 3MBA, 4ABA, and PyriBA functionalized substrates.
